# A Mobile Phone App Intervention to Promote Healthy Salt Intake Among Adults: Protocol for a Randomized Controlled Study

**DOI:** 10.2196/37853

**Published:** 2022-06-29

**Authors:** Milena Sia Perin, Thais São-João, Maria Cecília Bueno Jayme Gallani, Titilayo Tatiana Agbadje, Roberta Cunha Matheus Rodrigues, Marilia Estevam Cornélio

**Affiliations:** 1 Faculty of Nursing University of Campinas Campinas Brazil; 2 College of Nursing University of Rhode Island Kingston, RI United States; 3 Faculty of Nursing Laval University Quebec, QC Canada; 4 Canada Research Chair in Shared Decision Making and Knowledge Translation Laval University Quebec, QC Canada

**Keywords:** mHealth, intervention, sodium chloride, dietary, behavior change, mobile health

## Abstract

**Background:**

There is sound evidence associating high salt intake and a greater risk of cardiovascular and noncardiovascular diseases. High salt intake has been observed in several populations worldwide. Therefore, promoting healthier salt consumption has been encouraged as a low-cost strategy to reduce this risk factor. However, these strategies need to be sound, built on theoretical and methodological bases, and consider the target population’s context.

**Objective:**

This protocol aims to describe a mobile phone app intervention to promote healthy salt intake among adults.

**Methods:**

This is an experimental and longitudinal study protocol conducted in three modules. Module 1 refers to the planning of the intervention based on the Behaviour Change Wheel framework. Module 2 is the development of the mobile phone app intervention based on the date of module 1. In module 3, the intervention will be evaluated using a randomized controlled study, with three steps of data collection in a 2-month follow-up in a sample of 86 adults (43 participants for each group: the control group and intervention group) recruited from the primary health care centers of a Brazilian town. The discretionary salt intake questionnaire will assess salt consumption, the app usability will be assessed using the System Usability Scale, and psychosocial variables (habit, intention, and self-efficacy) will also be measured.

**Results:**

Recruitment began in October 2021, and the follow-up will end in August 2022. The results of this study are expected to be published in 2023.

**Conclusions:**

Results from this study will help people to control salt intake when cooking at home, will stimulate self-care, will work as an alternative or supportive method in the relationship between health care professionals and patients, and will contribute to implementing the app intervention to promote healthy salt intake on a large scale.

**Trial Registration:**

The Brazilian Clinical Trials Registry RBR-4s8qyyq; https://ensaiosclinicos.gov.br/rg/RBR-4s8qyyq

**International Registered Report Identifier (IRRID):**

DERR1-10.2196/37853

## Introduction

It is estimated that salt consumption is high worldwide [1,2], and there is sufficient evidence to associate high salt intake with the development and worsening of cardiovascular diseases [3-7]. A population-based study in a Brazilian town was conducted to characterize salt intake using biochemical and self-report methods, and according to sociodemographic and clinical variables, and found a mean of salt intake based on a 24-hour urine excretion of 10.5 g/day. Most of the salt is derived from the salt added during cooking [8]. It means consuming more than twice the daily recommendation of salt, which is less than 5 grams per day (2300 milligrams of sodium per day) [9,10], and it is the critical salt consumption behavior that should be reduced.

Plans and guidelines conducted by international and governmental agencies [7,11,12] encourage the promotion of a healthy intake of this nutrient in several countries, but changing people’s behavior is not a simple task for educators, researchers, health professionals, and those responsible for creating and implementing public policy strategies. Isolated educational campaigns or population awareness campaigns are insufficient. On the other hand, the results are effective when we associate behavior change interventions based on theoretical models in promoting healthy salt consumption [12-17].

A global systematic review [12] investigated 22 studies related to the impact of behavior change interventions to reduce salt consumption in the population and found that health education at the population level and awareness-raising interventions can improve salt-related behaviors and reduce their salt intake. In the analysis of the interventions developed based on methodological frameworks or models, all studies have shown success in improving the behavior or reduction of salt intake [12]. Thus, reducing salt intake is not a simple behavior to achieve, requiring more specific initiatives with the aid of consistent and effective methodological frameworks to change people’s dietary patterns [12,18,19].

One of the frameworks used to design a behavior change intervention is the Behaviour Change Wheel (BCW). The BCW is a validated synthesis of 19 frameworks of behavior change linked to a broad model that can be applied to any behavior in any setting [20]. At the center of the BCW is the Capability, Opportunity, Motivation, and Behaviour (COM-B) model, which recognizes that the behavior is a complex interaction of all these components, and its variant, the Theoretical Domains Framework (TDF), which synthesizes key theoretical constructs from the 33 behavior change theories [20]. The components of COM-B and the domains of TDF together give a more detailed diagnosis of the behavioral analysis. The BCW has been applied successfully in intervention development in various health-related contexts, including changing eating behaviors [19].

In addition to using a consistent tool to design the intervention, there is the choice of an appropriate way to deliver this intervention. Given advances in information technology, increasing smartphone users, easy internet access, and the use of mobile apps, health care app-based interventions have become a growing area for the promotion and self-monitoring of users’ health because of their cost-effectiveness by encouraging self-care [21], the flexibility in information delivery, and the breaking down of barriers that have been associated with traditional interactions [22,23].

As a result of a low cost and easy access, behavior change interventions through apps have increased considerably in several areas, such as physical activity–related behaviors [24,25], smoking [26], medication adherence [27,28], and depression [29,30] as well as chronic diseases such as diabetes [31] and asthma [32]. Based on this perspective, we set out to promote healthy salt consumption through the app.

Thus, the objective of this protocol is to describe a randomized pilot study aimed at assessing the efficacy of an app intervention to promote healthy salt intake based on the BCW in a Brazilian population and assessing the usability of the app.

## Methods

**Figure 1:**
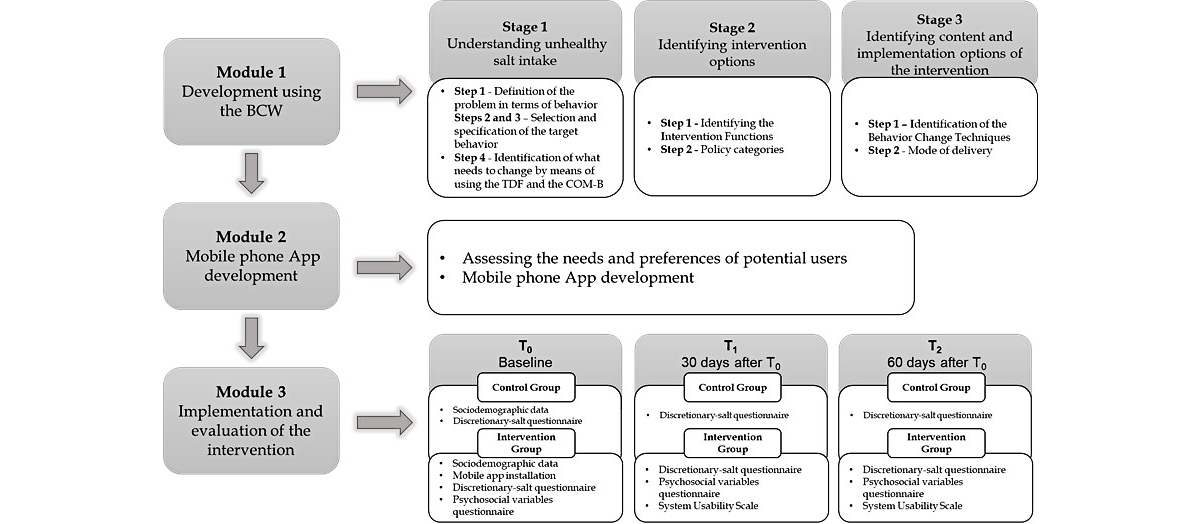
Intervention flow diagram. BCW: Behaviour Change Wheel; COM-B: Capability, Opportunity, Motivation, and Behaviour; TDF: Theoretical Domains Framework.

This study will be conducted in three modules: (1) intervention development using the BCW, (2) elaboration of the mobile phone app, and (3) evaluation of the intervention (Figure 1).

### Module 1: Intervention Development Using the BCW

Module 1 will follow the BCW stages: understanding unhealthy salt intake, identifying intervention options, and identifying the content and implementation options of the intervention [20].

#### Stage 1: Understanding Unhealthy Salt Intake

Stage 1 includes four steps: (1) definition of the problem in terms of behavior (unhealthy salt intake), (2) selection of the target behavior, (3) specification of the target behavior, and (4) identification of what needs to change using the TDF [33] and the COM-B model.

Steps 1, 2, and 3 will be developed based on previous studies on salt intake among the Brazilian population [8]. In step 4, the TDF and the COM-B model, proposed by Michie and collaborators [20], will be used to identify the behavioral determinants targeted by the intervention. The TDF is an integrative framework developed from a synthesis of psychological theories to provide a comprehensive approach to identifying determinants of behavior [33].

#### Stage 2: Identifying Intervention Options

The first step is called intervention functions. From these functions, it is possible to assess which of these types of intervention give the best results and may be able to change the behavior. The intervention functions will be selected after identifying TDF and COM-B components. There are nine functions described in the BCW: (1) education, (2) persuasion, (3) incentivization, (4) coercion, (5) training, (6) restriction, (7) environmental restructuring, (8) modeling, and (9) enablement.

After selecting functions, we will apply the Affordability, Practicability, Effectiveness/cost-effectiveness, Acceptability, Side-effects/safety, Equity (APEASE) criteria that is used for designing and evaluating interventions [20]. In our study, the APEASE criteria will be by an experts committee formed by two professionals with experience in theories of behavior change, development, and implementation of the intervention; a research professional with knowledge of the BCW guide; a clinician with experience in patient counseling for salt consumption; and a researcher with knowledge of salt consumption and behavior change techniques (BCTs) [34].

The next step in this stage is the policy categories. This step refers to the inclusion of policy makers in the implementation and execution of an intervention. This step is optional. Due to the difficulty of contacting these policy makers in the research development region, they will not participate in this study.

#### Stage 3: Identifying Content and Implementation Options of the Intervention

In stage 3, BCTs and the mode of delivery of the intervention are defined.

In step 1, after identifying the intervention functions, the expert committee will determine the most appropriate BCTs based on the intervention functions selected in stage 2. A BCT is an “observable, replicable, and irreducible component of an intervention designed to change behavior and a postulated active ingredient within the intervention” [20]. This step also requires the application of the APEASE criteria to select the BCTs that will be used in the intervention.

The last step corresponds to the mode of delivery. Driven by recent advances in the use of mobile apps and their use as a health promotion tool delivering beneficial results to users in many areas [24-32] and the current scenario of a pandemic caused by SARS-CoV-2, in which it is necessary to socially distance, we will choose to use the mobile phone app as a remote administration mode. For this choice, a delivery taxonomy was consulted [20].

### Module 2: Mobile Phone App Development

Module 2 aims to develop the mobile phone app, which requires data collection from participants who are potential users of the app.

#### Study Design

To develop the mobile phone app, we will conduct an exploratory study aiming at assessing the needs and preferences of potential users regarding the use of an app for reducing salt.

Potential users will be interviewed to assess their preferences regarding receiving the intervention by the app in an attempt to absorb this empirical reality, which is continually changing, either due to sociocultural diversity, epidemiological dynamics, or technological changes [35].

#### Participants and Recruitment

Potential users were defined as any person interested in using an app for reducing salt. To be eligible, they should be aged between 20 and 59 years (classification of the Brazilian Institute of Geography [36] for adults, the same age group used in a previous study to choose the target behavior), speak Portuguese, have a mobile phone that supports mobile apps, have skill in handling a cell phone, and consent to participate in the study.

Participants will be recruited from a primary health center in an urban Brazilian town, Artur Nogueira, with 56,247 inhabitants, located in a metropolitan region of Sao Paulo, Brazil [36]. A research assistant will be present in the waiting room of the Basic Health unit of the clinical site to meet patients and offer them the opportunity to participate in the project.

#### Data Collection

We will conduct semistructured interviews with potential users to explore participants’ needs (eg, content) regarding the mobile app, their preferences (eg, features and settings) regarding the mobile app, their current use (if any) of or search for health care apps, and their perception of an app for controlling and monitoring salt addition in meal preparation, as we are proposing.

At the beginning of the interview, each participant will be invited to sign a consent form. The interviews will last 30 to 45 minutes and will be recorded.

#### Sample Size

The interviews will be conducted with a convenience sample of potential users of the app. According to Guest and collaborators [37], data saturation occurred within the first 12 interviews. Considering this, we planned to collect data from at least 12 participants while ensuring data saturation.

#### Data Analysis

Audios will be recorded in transcripts and used to extract the information related to factors cited in the Data Collection section and any other relevant information from the interviews. The extracted data will be processed and used to guide the following mobile app development phase.

#### Procedure for Mobile Phone App Development

The mobile app will be developed based on the previous phases/steps and qualitative exploration. We will follow the guidelines for describing health interventions using mobile phones (the mHealth evidence reporting and assessment checklist [38]). The app will be developed by a software engineer using the Android operating system, and it will be written in JavaScript using the React Native framework. After its elaboration, the mobile app will be evaluated by experts to assess its content and layout.

### Module 3: Implementation and Evaluation of the Intervention

#### Study Design

To evaluate the efficacy of the intervention, we will conduct a randomized controlled study, with three waves of data collection in a 2-month follow-up. The SPIRIT (Standard Protocol Items: Recommendations for Interventional Trials) guideline was followed in the description of this module.

#### Eligibility Criteria

The study population is adults living in a Brazilian town (described in module 2). To be eligible to participate in this study, they should be aged between 20 and 59 years, have access to a mobile phone that supports the mobile app, speak Portuguese, and consent to participate in the study.

#### Sample Size and Recruitment

According to the quantitative outcomes, the sample size was calculated considering the objective of comparing the two groups (control group [CG] and intervention group [IG]) in the three periods of time. The methodology of a sample calculation for a repeated-measures ANOVA model was considered to perform this calculation. In this calculation, a significance level of 5%, a test power of 80%, and an effect size of 0.25 were assumed, which, according to Cohen [39], can be considered a medium degree effect size. The first calculation resulted in a sample of 86 participants, but a 20% rate for possible losses was considered, which resulted in a sample of 108 participants (54 participants per group).

Recruitment will occur in primary health care centers of a Brazilian town, with the help of the community health agent of the unit that is linked to each resident of its microarea; the individual will be approached and will invite a resident at home who meets the inclusion criteria to participate in the study. Participants will be selected in a randomized way and will be divided between groups (IG and CG), considering a randomization scheme by blocks of random size. The randomization scheme was generated online [40].

#### Outcomes

##### System Usability Scale

The scale was created in 1986 by Brooke [41] to measure the usability of products and services, including hardware, software, mobile devices, websites, and applications. It consists of a 10-item questionnaire with five response options for respondents that range from *strongly agree* to *strongly disagree* [41]. This scale will be used to assess the usability of the app.

#### Primary Outcome

##### Discretionary Salt Questionnaire

The discretionary salt questionnaire was validated in a previous study [42,43] on the Brazilian population. Participants will report their regular monthly salt consumption (number of 1 kg packages of salt consumed per month), the number of persons who ate meals at home, the age of each person, and the number of meals that each person eats per week at home to correct the salt consumption per person. Children younger than 3 years will not be considered in the calculation, and the meals of children younger than 10 years will be regarded as half meals. The following steps will be used to calculate the salt intake per person: divide the amount of salt (grams) used per month at home by 30 and multiply the quotient by 7 (=grams of weekly salt intake at home), divide this amount of weekly salt intake by the total number of weekly meals (=grams of salt intake/meal), multiply the amount of salt used per meal by the number of meals eaten per week by the participant, and divide the total weekly salt intake of the participant by 7, resulting in the estimated quantity of individual daily added salt. The discretionary salt questionnaire will be used to evaluate the efficacy of the intervention.

#### Secondary Outcomes

##### Habit

The first psychosocial variable interferes with the adoption of behavior and will be evaluated through the mean of 10 items [44] assessed on a 5-point scale (1, definitely not, to 5, definitely yes). The guiding phrase for the investigation of the habit will be “Using more than one level teaspoon of salt/day (ie, more than 3 g of salt) during cooking is something that I do...” The items will be “I do it frequently”; “I do it automatically”; “I do it without having to remember to do it consciously”; “If I don’t, it make me feel strange”; “I do it without thinking”; “It would take effort not to do it”; “It is part of my day-to-day”; “I start doing it without realizing that I’m already doing it”; “I would find it difficult not to do”; and “I've been used to doing this for a long time.” The higher the response score, the greater the individual’s favorability of not restricting the amount of salt used during cooking.

##### Self-Efficacy

The second psychosocial variable will be estimated through the mean of 5 items [44] rated on a 5-point scale (1, completely disagree, 5, completely agree. The higher the score, the greater the perception of self-efficacy to perform the behavior. The statements were “I trust my ability to use 1 level teaspoon of salt a day during cooking,” “I can use 1 level teaspoon of salt a day during cooking,” and “I am sure that I can use 1 level teaspoon of salt a day during cooking.”

##### Intention

The last psychosocial variable will be estimated through the mean of 6 items [44] rated on a 5-point scale. A high score indicates a high intention to perform the behavior. “I am planning to use 1 level teaspoon of salt a day during cooking” (1, definitely not, 5 definitely yes), “I will try to use 1 level teaspoon of salt a day during cooking” (1, definitely not, 5, definitely yes), “I want to use 1 level teaspoon of salt a day during cooking” (1, I totally disagree, 5, I totally agree). “I hope to use 1 level teaspoon of salt a day during cooking” (1, unlikely, 5, very likely), “How likely are you to use 1 level teaspoon of salt a day during cooking” (1, unlikely, 5, very likely).

#### Data Collection

Data collection will be performed in three steps (T0, T1, and T2), considering a period of 1 month between them. At the first step (T0), participants will be informed about the research, the study’s relevance, and the need to return to the next meeting, and will sign the informed consent form. Sociodemographic data, the discretionary salt questionnaire, habit, self-efficacy, and intention will be evaluated; the mobile app will be installed on their phone and guidance will be given on using the mobile app. Both groups will be assessed at T1 (1 month after T0) and T2 (1 month after T1) for the discretionary salt questionnaire, habit, self-efficacy, and intention variables. The usability of the mobile app (System Usability Scale) will be evaluated in the intervention group in T1 and T2. At the end of the study, participants in the CG will have the right to have the app installed on their cell phones if they wish and receive instructions on how to use it.

#### Data Analysis

Qualitative data will be described using frequencies and percentages, and quantitative data as measures of central tendency (mean and median) and dispersion (SD, maximum, and minimum). Correlation tests (Spearman or Pearson coefficient) will test the relation between habit and self-efficacy with intention at T0. Regression analysis using generalized linear models will be used to verify if habit, self-efficacy, and intention at T0 predict the discretionary salt questionnaire at T1 and T2.

To evaluate the efficacy of the intervention, comparisons between groups and times about the discretionary salt questionnaire will be performed using a linear regression model via generalized estimating equations modeling [45].

A significance level of 5% will be adopted. Statistical analyses will be performed using SAS, version 9.4 (SAS Institute).

### Ethics and Dissemination

Participants from the data collection of phases 1 of module 2 (user needs assessment) and module 3 (implementation and evaluation of intervention) will provide written informed consent. At this moment, complete and adequate information about the study’s nature, purpose, and possible risks and benefits are given to the participants, confirming that they understand the study requirements according to the local ethics committee that approved the study (Process No. 10937419.0.0000.5404).

## Results

### Module 1

The development of module 1 has been completed, and the results are described in the following sections.

Recruitment began on October 1, 2021. The trial will complete the recruitment phase in June 2022, and the follow-up phase will end in August 2022. The publication of results is anticipated in 2023.

All steps of stage 1 (understanding unhealthy salt intake) have been entirely detailed.

### Step 1 of Stage 1: Definition of the Problem in Terms of Behavior (Unhealthy Salt Intake)

Due to the high salt consumption evidenced in the previous study in the same town where this project will be developed and considering the health risks and harms resulting from this consumption, there was a need to promote the healthy consumption of this nutrient not only to specific groups but also at the population level.

### Step 2 of Stage 1: Selection of the Target Behavior

Perin et al’s [8] study also demonstrated that, regarding the sources of salt intake, 24% came from the intrinsic sodium assessed from a 24-hour dietary recall and 7.3% from ultra-processed foods with high sodium content, and the addition of salt in food preparation was the behavior that contributed to 59.1% of the overall salt intake in the population. Thus, reducing salt addition in food preparation was selected as the target behavior of the intervention.

### Step 3 of Stage 1: Specification of the Target Behavior

In this step, it is essential to have a specific behavior because, according to Michie and collaborators [20], the clearer the behavior, the better your behavioral analysis. Thus, the salt added to food preparation will be monitored daily at home by the person or by the person helping them cook.

### Step 4 of Stage 1: Identification of What Needs to Change (the TDF and COM-B Model)

First, we selected the TDF using the determinants described in Cornélio et al’s [46] study. This study applied an extended version of the Theory of Planned Behavior among Brazilian individuals with hypertension and found that self-efficacy and habit were significant determinants of intention, explaining 62% of the variability of intention to add less than 4 g of salt per day in food preparation, and the intention, on the other hand, explained 22% of the variability of this behavior [46]. Thereby, the domains of the TDF were mapped from these three determinants—intention, self-efficacy, and habit—to identify what needed to change. In the second process, we identified the components of the COM-B model from the TDF domains, which could be physical capability, psychological capability, social opportunity, physical opportunity, reflective motivation, and automatic motivation. The findings are described in Table 1.

From this process, we are capable of developing stage 2: identifying intervention options.

**Table 1:** Linking of the determinant of the TPB^a^, the domain of the TDF^b^, and the COM-B^c^ model based on Michie and collaborators [20].

Determinant of TPB (based on Cornélio et al [46])	Domain of TDF (definition)	Component of COM-B (definition)
Intention	Intentions (a conscious decision to perform a behavior or a resolve to act in a certain way)	Reflective motivation (reflective processes involving plans [self-conscious intentions] and evaluations [beliefs about what is good and bad])
Self-efficacy	Beliefs about capabilities (acceptance of the truth, reality, or validity about an ability, talent, or facility that a person can put to constructive use)	Reflective motivation
Habit	Behavioral regulation (anything aimed at managing or changing objectively observed or measured actions)	Psychological capability (knowledge or psychological skills, strength, or stamina to engage in the necessary mental process)

^a^TPB: theory of planned behavior.

^b^TDF: Theoretical Domains Framework.

^c^COM-B: Capability, Opportunity, Motivation, and Behaviour.

### Step 1 of Stage 2: Intervention Functions

The intervention functions that met the APEASE criteria were education (increasing knowledge or understanding), persuasion (using communication to induce positive or negative feelings, or to stimulate action), incentivization (creating an expectation of reward), modeling (providing an example for people to aspire to or imitate), enablement (increasing means or reducing barriers to increase capability or opportunity), and training (imparting skills).

Finally, after identifying intervention functions, we achieved stage 3: identifying content and implementation options.

### Step 1 of Stage 3: Behavior Change Techniques

The selection process of the techniques resulted in 31 BCTs after removing duplicates. In conclusion, after applying APEASE criteria, we had a list of 16 BCTs: goal setting (behavior), problem solving, goal setting (outcome), action planning, commitment, feedback on behavior, self-monitoring of behavior, social support (unspecified), social support (practical), instruction on how to perform the behavior, information about health consequences, demonstration of the behavior, prompt/cues, behavioral practice/rehearsal, credible source, and adding objects to the environment.

## Discussion

### Expected Findings

We have described our innovative protocol for an intervention to promote healthy salt intake using the BCW, a guide to designing behavior change interventions. The design of this protocol study has considered the evidence on the efficacy of changing eating behaviors using the BCW [19], the advances in the use of the technology of mobile apps in various areas of health (mainly in eating behaviors [47]), and the dietary recommendations to reduce salt intake for the prevention of cardiovascular diseases [48].

To our knowledge, there is only one study available in the literature that evaluated the effect of an app intervention to reduce salt consumption using BCW; however, the study was focused on people with arterial hypertension and not on the general population [49].

We expected that this theory- and evidence-based mobile app intervention would effectively promote healthy salt intake when cooking at home since this behavior is the primary source of consumption of this nutrient among the Brazilian population. We also hope that mobile apps as the mode of delivering the intervention will encourage and facilitate people’s self-care and work as an alternative or supportive method in the relationship between health care professionals and patients. Finally, it is expected that the study will inform the potential scalability and transferability of this intervention for achieving a broader public health impact.

The next phase of this research is to complete the development of module 2, the semistructured interviews with the potential users of the mobile app, and the mobile app. After that, we will implement and evaluate the intervention (module 3) using a randomized controlled study.

The results of this study will be published in peer-reviewed scientific journals and presented at scientific events in the thematic area. They will also be communicated to research participants and the public involved in the study through face-to-face presentations at the health services participating in the research.

### Strengths and Limitations

Our protocol intervention has some strengths. First, a behavioral analysis guided by the BCW was carried out to gain a theoretical understanding of the specific behavior of salt consumption and the factors that influence it. Besides that, we involve potential users in the development process, which will make it possible to tailor the app to the target audience. This will allow a robust design to be created to increase the efficacy of the intervention. Last, this is the first time that an intervention will be developed to help monitor salt addition in meal preparation for the Brazilian population through a mobile app. A new approach in this area, regarding the recent advances in the field of mobile apps, has been showing its use as a health promotion tool with beneficial results in other areas, including depression, diabetes, medication adherence, respiratory diseases, and physical activities [24,25,27-32].

This study also has limitations. First, we did not consider the political levers in developing our intervention because we did not have access to them. Second, the choice of the intervention delivery mode using a mobile app was based on the evidence of success in previous studies.

### Conclusions

This protocol details a theory- and evidence-based intervention to promote healthy salt intake behavior among Brazilian adults. The steps of the BCW were successfully applied through a systematic process to identify the behavioral determinants and select the intervention options and the BCTs. A mobile phone app was chosen as the mode of intervention delivery.

## Abbreviations

APEASE: Affordability, Practicability, Effectiveness/cost-effectiveness, Acceptability, Side-effects/safety, Equity
BCT: behavior change technique
BCW: Behaviour Change Wheel
CG: control group
COM-B: Capability, Opportunity, Motivation, and Behaviour
IG: intervention group
SPIRIT: Standard Protocol Items: Recommendations for Interventional Trials
TDF: Theoretical Domains Framework

## Data Availability

Due to its proprietary nature and ethical concerns, supporting data cannot be publicly available. The supporting data of this study will be stored in the Research Data Repository of the University of Campinas [50]. Data may also be available after publication of the study results and upon reasonable request to the corresponding author.
